# Application of Untargeted Metabolomics to Reveal the Taste-Related Metabolite Profiles during Mandarin Fish (*Siniperca chuatsi*) Fermentation

**DOI:** 10.3390/foods11070944

**Published:** 2022-03-25

**Authors:** Yueqi Wang, Shi Nie, Chunsheng Li, Huan Xiang, Yongqiang Zhao, Shengjun Chen, Laihao Li, Yanyan Wu

**Affiliations:** 1 Key Laboratory of Aquatic Product Processing, Ministry of Agriculture and Rural Affairs of The People’s Republic of China, National R&D Center for Aquatic Product Processing, South China Fisheries Research Institute, Chinese Academy of Fishery Sciences, Guangzhou 510300, China; wangyueqi@scsfri.ac.cn (Y.W.); nieshi1003@163.com (S.N.); lichunsheng@scsfri.ac.cn (C.L.); skyxianghuan@163.com (H.X.); zhaoyq@scsfri.ac.cn (Y.Z.); chenshengjun@scsfri.ac.cn (S.C.); laihaoli@163.com (L.L.); 2 Co-Innovation Center of Jiangsu Marine Bio-Industry Technology, Jiangsu Ocean University, Lianyungang 222005, China; 3 Collaborative Innovation Center of Seafood Deep Processing, Dalian Polytechnic University, Dalian 116034, China

**Keywords:** mandarin fish fermentation, metabolomic profile, water-soluble metabolite, fermentation, quality control, *Siniperca chuatsi*

## Abstract

Spontaneous fermentation is a critical processing step that determines the taste quality of fermented mandarin fish (*Siniperca chuatsi*). Here, untargeted metabolomics using ultra-high-performance liquid chromatography coupled with Q Exactive tandem mass spectrometry was employed to characterize the taste-related metabolite profiles during the fermentation of mandarin fish. The results demonstrated that the taste profiles of mandarin fish at different stages of fermentation could be distinguished using an electronic tongue technique. Sixty-two metabolites, including amino acids, small peptides, fatty acids, alkaloids, and organic acids, were identified in fermented mandarin fish samples. Additional quantitative analysis of amino acids revealed glutamic acid and aspartic acid as significant contributors to the fresh flavor. Furthermore, the Kyoto Encyclopedia of Genes and Genomes pathway enrichment analysis revealed that amino acid metabolism was the dominant pathway throughout the fermentation process. This study provides a scientific and theoretical reference for the targeted regulation of the quality of fermented mandarin fish.

## 1. Introduction

Fermentation is an ancient food processing technique that is often employed to preserve food and improve its appearance and taste [1]. Mandarin fish is a common carnivorous fish that is widely distributed in China, Vietnam, Korea, Japan, and other neighboring countries [2]. In China, it is an important high-quality freshwater farmed fish, with an annual production of over 300,000 tons [3]. Fermented mandarin fish is a popular traditional Chinese food, owing to its unique flavor and high concentration of dietary proteins and lipids [4]. Typically, fermented mandarin fish is produced by natural fermentation in the presence of a low concentration of salt and spices. Yang et al. [5] found that metabolites such as glutamic acid, valine, and alanine play an important role in determining the flavor of mandarin fish. A series of complex metabolic activities occur during the fermentation process, mainly involving the degradation of nutrients, and a variety of volatile and nonvolatile secondary metabolites, which enrich the flavor of food, are generated via various biochemical metabolic pathways [6].

Taste develops as a result of the binding of water-soluble taste-presenting substances to human taste receptors, and it is an important indicator of the quality of fermented mandarin fish [7]. The electronic tongue is based on the biological mechanism of taste perception. The sensor array in the electronic tongue system is the biological equivalent of a tongue; it collects information of the electrical signals of different chemical substances and feeds them into a computer. It uses multidimensional statistical analysis to differentiate different substances and provide sensory information of each of them. Thus, the electronic tongue can be used to differentiate the taste of fermented mandarin fish [8]. The taste components of fermented fish are mainly water-soluble small-molecule compounds, including nitrogenous components (free amino acids, small peptides, nucleotides, and organic bases), non-nitrogenous components (sugars, organic acids, and inorganic salts), and other substances. The metabolites and mechanisms involved in the fermentation of mandarin fish are very complex, and the fermentation process is regulated by multiple factors, including temperature [9], salinity [10], and environmental microorganisms [11], which interact with each other by modulating numerous metabolic pathways [12]. Therefore, the use of traditional methods of food analysis, such as high-performance liquid chromatography (HPLC) and gas chromatography, to analyze a single chemical component or a class of chemical components leads only to a limited understanding of the fermentation process; it does not provide an overall understanding of the basic characteristics of traditional fermented foods.

Metabolomics is a rapidly emerging high-throughput analysis technique that provides a more comprehensive approach to discover direct relationships between metabolites and metabolic pathways by linking metabolite levels to phenotypic changes in organisms [13]. Untargeted metabolomics has been widely used in the study of fermented foods, such as coffee [14], tea [15], and fish sauce [16]. Although metabolomic-based strategies have been effective in elucidating the metabolite profiles of some fermented foods, to the best of our knowledge, no study has used these methods to characterize the changes in metabolites during the fermentation of mandarin fish.

Previously, we evaluated the succession of the autochthonous microbiota in the process of flavor formation during the fermentation of mandarin fish [4]. We hypothesized that metabolomic approaches could be used to characterize the dynamics of key metabolites that determine the taste of fermented mandarin fish. In this study, untargeted metabolomics using ultra-HPLC (UHPLC) coupled with Q Exactive tandem mass spectrometry (MS/MS) was applied to systematically assess the metabolite profiles and taste quality of fermented mandarin fish. This study is expected to provide a scientific theoretical basis for targeted regulation of taste quality in the fermented fish industry.

## 2. Materials and Methods

### 2.1. Sample Preparation

Mandarin fish was fermented as reported in our previous study [4]. Mandarin fish was supplied by a fish processing plant in Anhui Province, China. The frozen fish were thawed, gutted, cleaned, and placed neatly in a fermentation tank; thereafter, 5% edible coarse salt and 0.02% spices (pepper and chili powder) were added, and fermentation was conducted under natural conditions (20 ± 5 °C and a relative humidity of 45% ± 15%). Samples were collected in five replicates each on days 0, 4, 8, and 12 (hereinafter referred to as groups 0D, 4D, 8D, and 12D, respectively) of the fermentation process. The collected samples were immediately stored in food-grade sterile bags at −18 °C for further analysis.

### 2.2. Electronic Tongue Analysis

Electronic tongue was used according to He’s method [17], with some modifications. The taste profiles of the fermented mandarin fish samples were determined at different fermentation stages using an ASTREE electronic tongue (Alpha MOS, Toulouse, France). The electronic tongue cannot detect the intensity signal of the concentration of a specific compound in solution, but it can reflect the overall taste characteristics of the food. Premoistening was required before detection to restore the activity of the sensor membrane. The seven sensors (AHS, PKS, CTS, NMS, CPS, ANS and SCS) were soaked in 30 mM KCl + 0.3 mM tartaric acid for 24 h; the electrode was soaked in 3.3 M KCl. The electronic tongue integrates the potential difference signals from seven sensors, and through further analysis, it can directly derive the relative sourness, freshness, and saltiness intensity of each sample to discover the taste differences between different samples. For sample processing, 100 mL of ultrapure water was added to 10 g of chopped fermented mandarin fish to extract flavoring substances. The sample was thoroughly stirred with a magnetic stirrer and centrifuged at 10,000× *g* for 10 min at 4 °C; the supernatant was poured into the electronic tongue injection cup, and the measurement was performed at approximately 25 °C. The data acquisition time was 120 s. The 120-s response value of each sensor was selected as the raw data of the electronic tongue. The sensors were cleaned with deionized water for 10 s after each measurement. Five parallel experiments were performed for each sample group, with the first two data cycles deleted, and data from three cycles were used for analysis.

### 2.3. Extraction of Metabolites

Fermented mandarin fish samples (50 mg) were added to 1 mL of the extraction solution (methanol:acetonitrile:water = 2:2:1, *v*/*v*/*v*), followed by grinding in a grinder (JXFSTPRP-24; Jingxin Co., Ltd., Shanghai, China) at 35 Hz for 4 min and sonication in an ice water bath for 5 min. The grinding and ultrasonic treatments were repeated two or three times. The samples were then incubated for 1 h at −40 °C and centrifuged at 12,000× *g* for 15 min at 4 °C. The supernatant was used for further analysis. To verify the reproducibility and stability of instrumental analysis, all samples were subjected to five tie-breaking experiments.

### 2.4. Metabolomic Analysis Using UHPLC-QE-MS/MS

A Vanquish UHPLC system (Thermo Fisher Scientific, Waltham, MA, USA) was used with an ACQUITY UPLC BEH amide column (2.1 mm × 100 mm, 1.7 μm; Waters Corp., Milford, MA, USA) to separate the metabolites. Mobile phase A comprised 0.025 M ammonia and 0.025 M ammonium acetate, and phase B comprised pure acetonitrile. Gradient elution was performed as follows: 0–0.5 min, 95% B; 0.5–7 min, 95–65% B; 7–8 min, 65–40% B; 8–9 min, 40% B; 9–9.1 min, 40–95% B; and 9.1–12 min, 95% B. The mobile phase flow rate was 0.5 mL/min; the column temperature was 30 °C; the sample tray temperature was 4 °C; and the injection volume was 3 μL. Quality control (QC) samples were added to the samples to monitor the stability of the system and ensure reliable experimental data. A Thermo Q Exactive HFX mass spectrometer (Thermo Fisher Scientific), capable of primary and secondary MS data acquisition, was used. The electrospray ionization (ESI) conditions were as follows: sheath gas flow rate, 30 Arb; auxiliary gas flow rate, 25 Arb; capillary temperature, 350 °C; full MS resolution, 60,000; MS/MS resolution, 7500; collision energy, 10/30/60 in normalized collision energy mode; and spray voltage, 3.6 kV (positive) or −3.2 kV (negative).

### 2.5. Identification and Quantification of Free Amino Acids

Free amino acids were extracted using the methods proposed by Zhang et al. [18]. To the samples (1.000 ± 0.001 g) collected at different fermentation periods, 15 mL of 5% trichloroacetic acid was added and the samples were homogenized and sonicated. The samples were then incubated at 4 °C for 2 h and centrifuged at 12,000× *g* for 15 min at 4 °C. The pH of the supernatant (5 mL) was then adjusted to 2.0 by adding 6 mol/L or 1 mol/L NaOH; the supernatant was then diluted to 10 mL with ultrapure water in a volumetric flask. The sample solution was then passed through a 0.22-micrometer organic phase filter membrane and injected into a sample vial of an amino acid analyzer (L-8800; Hitachi Co., Ltd., Tokyo, Japan). Each sample was measured three times. The free amino acid results are expressed as mg/100 g sample.

### 2.6. Metabolic Pathway Enrichment Analysis

The Kyoto Encyclopedia of Genes and Genomes (KEGG) pathway database (http://www.kegg.jp/kegg/pathway.html, accessed on 14 January 2022) was used to analyze metabolic pathways during the fermentation of mandarin fish. The significantly different metabolites identified in the mandarin fish samples were mapped to the KEGG pathways, and the pathways were enriched and analyzed to find metabolic pathways with the highest correlation with differential metabolites.

### 2.7. Data Processing and Statistical Analysis

Data were processed and examined using SIMCA software V16.0.2 (Sartorius Stedim Data Analytics AB, Umeå, Sweden). The significant differences and Pearson correlation coefficients were assessed using SPSS 19.0 software (IBM Corp., Armonk, NY, USA). Multivariate analysis included principal component analysis (PCA) and orthogonal partial least squares discriminant analysis (OPLS-DA), and the metabolite values of 0D versus 4D, 4D versus 8D, and 8D versus 12D were screened based on *p* < 0.05 and the variable importance of projection (VIP) values. TBtools version 1.082 (Guangdong, China) was used to generate a hierarchical clustering heatmap.

## 3. Results and Discussion

### 3.1. Analysis of the Taste Profiles during the Fermentation of Mandarin Fish

**Figure 1:**
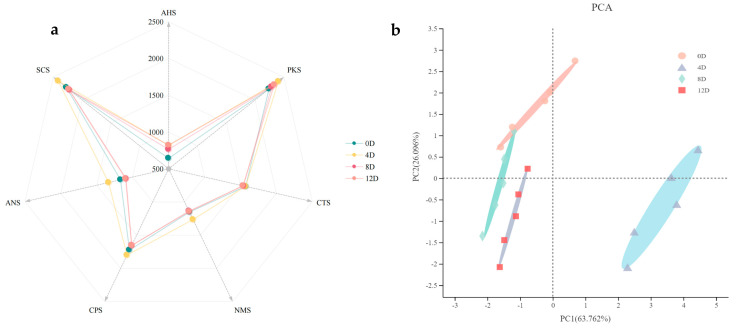
Electronic tongue radargram analysis spectra (**a**) and the scatter plot for principal component analysis (PCA) (**b**) of mandarin fish samples at different fermentation stages.

The electronic tongue can sensitively discriminate the taste of a sample and make objective judgments of the taste based on a multi-sensor array that simulates the human tongue [19]. The radar diagram (Figure 1a) illustrates the response of the sensors of the electronic tongue to substances that impart taste to mandarin fish, during different fermentation periods. Taste differences during fermentation were mainly reflected by the AHS, NMS, and ANS sensors (Figure 1a). Throughout the fermentation process, the AHS response values were always the lowest, and the SCS and PKS response values were higher than those of the other sensors. The higher SCS response values may have been due to the ability of salts and acids to enhance each other at moderate concentrations and the ability of acidity to enhance bitterness under certain conditions [20]. As the fermentation process progresses, proteins are gradually broken down into amino acids and small peptides, which can also lead to an increase in bitterness [21]. A previous study that investigated fermented sausages found that sour response values were generally low throughout the fermentation process, whereas bitter response values were relatively high [22]. The NMS, CPS, SCS, and ANS responses reached maximum values on day 4 of fermentation, whereas the PKS and CTS response values showed small differences in taste at different fermentation stages, indicating that fermentation time had no effect on PKS and CTS values.

Subsequently, the PCA was used to further identify the differences in taste profiles based on the electrical signal as the fermentation progressed (Figure 1b). Principal components PC1 and PC2 explained 63.8% and 26.1%, respectively, totaling 89.9% of the variation in taste among the different fermentation periods of the samples. The results demonstrated that the electronic tongue could clearly distinguish the taste profiles at different fermentation stages. The samples fermented for 0, 4, 8, and 12 days were mainly concentrated in the second, fourth, and third quadrants, indicating that the overall taste of mandarin fish changed as fermentation progressed.

### 3.2. Multidimensional Statistical Analysis of the Metabolite Profiles during the Fermentation of Mandarin Fish

**Figure 2:**
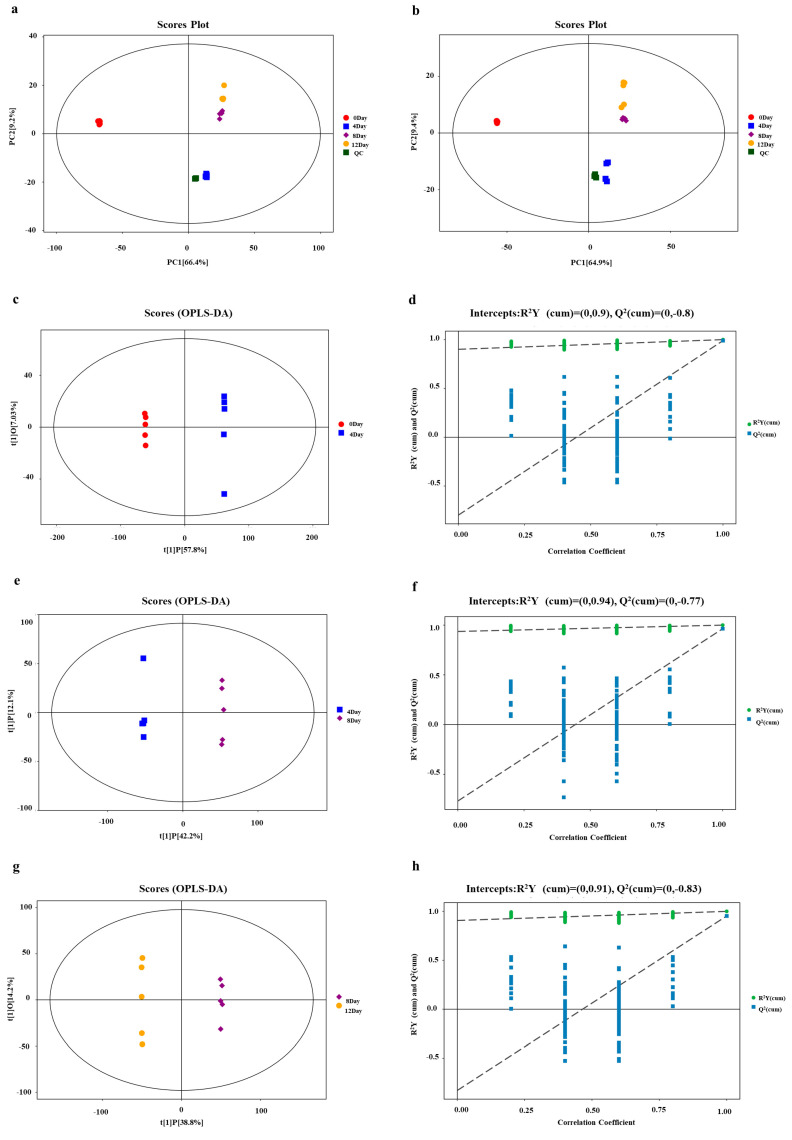
Principal component analysis (PCA) score plots of all samples of fermented mandarin fish and the quality control (QC) sample in the ESI^+^ (**a**) and ESI^−^ (**b**) modes. Orthogonal partial least squares discriminant analysis score scatter plots in the positive ion mode: (**c**) 0D versus 4D; (**e**) 4D versus 8D; and (**g**) 8D versus 12D. Displacement test results in the positive ion mode: (**d**) 0D versus 4D; (**f**) 4D versus 8D; and (**h**) 8D versus 12D.

UHPLC-QE-MS/MS was applied to identify metabolite profiles in different fermentation periods. A total of 18,331 metabolite ion signatures were extracted, among which 10,010 and 8321 peaks were extracted in the positive and negative ion modes, respectively. All samples were clustered using the PCA within a 95% confidence interval (Figure 2a,b). The QC samples (green) in the ESI^+^ and ESI^−^ modes were clustered closely, and the samples from the four fermentation stages were located in three different quadrants, indicating good stability of the instrumental analysis system and high data reliability during the detection process [23]. These results explained 75.6% and 74.3% of the differences between samples in the ESI^+^ and ESI^−^ modes, respectively, which could better distinguish the samples from different fermentation periods. The samples collected on days 4, 8, and 12 of fermentation were on the negative half-axis of PC1, whereas those collected on day 0 of fermentation were within the positive half-axis of PC1 (Figure 2a,b). There were large differences between the unfermented samples (day 0) and those at different fermentation stages, indicating that the metabolites in mandarin fish underwent large changes during the fermentation process.

To obtain more reliable information on the differences in metabolites between the groups, the OPLS-DA analysis was performed to further analyze and predict the data. The results indicated that the model distinguished the fermentation groups 0D versus 4D, 4D versus 8D, and 8D versus 12D. As fermentation progressed, the changes in metabolites were greater in the initial fermentation stages than in the late fermentation stages. The model was tested using seven-fold cross-validation, and then judged on the basis of the obtained *R*^2^Y and *Q*^2^ values. The model evaluation parameters are shown in Figure 2c–h. Categorical variables *R*^2^Y were greater than 90% for the three model groups, 0D versus 4D, 4D versus 8D, and 8D versus 12D. Moreover, the *Q*^2^ value was >0.5, indicating that the model was stable and reliable, with good predictability. Furthermore, the intercept of the Q2 regression line was less than 0.05, indicating that the OPLS-DA model was not overfitting and that there was differential metabolism between these three groups that could be reliably used to screen for differences in metabolites.

### 3.3. Screening for Differential Metabolites

Three groups (0D versus 4D, 4D versus 8D, and 8D versus 12D) of metabolites in the positive and negative ion modes were screened according to VIP > 1.3 and *p* < 0.05. Finally, 48, 50, and 32 differential metabolites were identified by screening the three pairs (0D versus 4D, 4D versus 8D, and 8D versus 12D, respectively). A correlation matrix was used to reveal the interrelationships between the metabolites.

**Figure 3:**
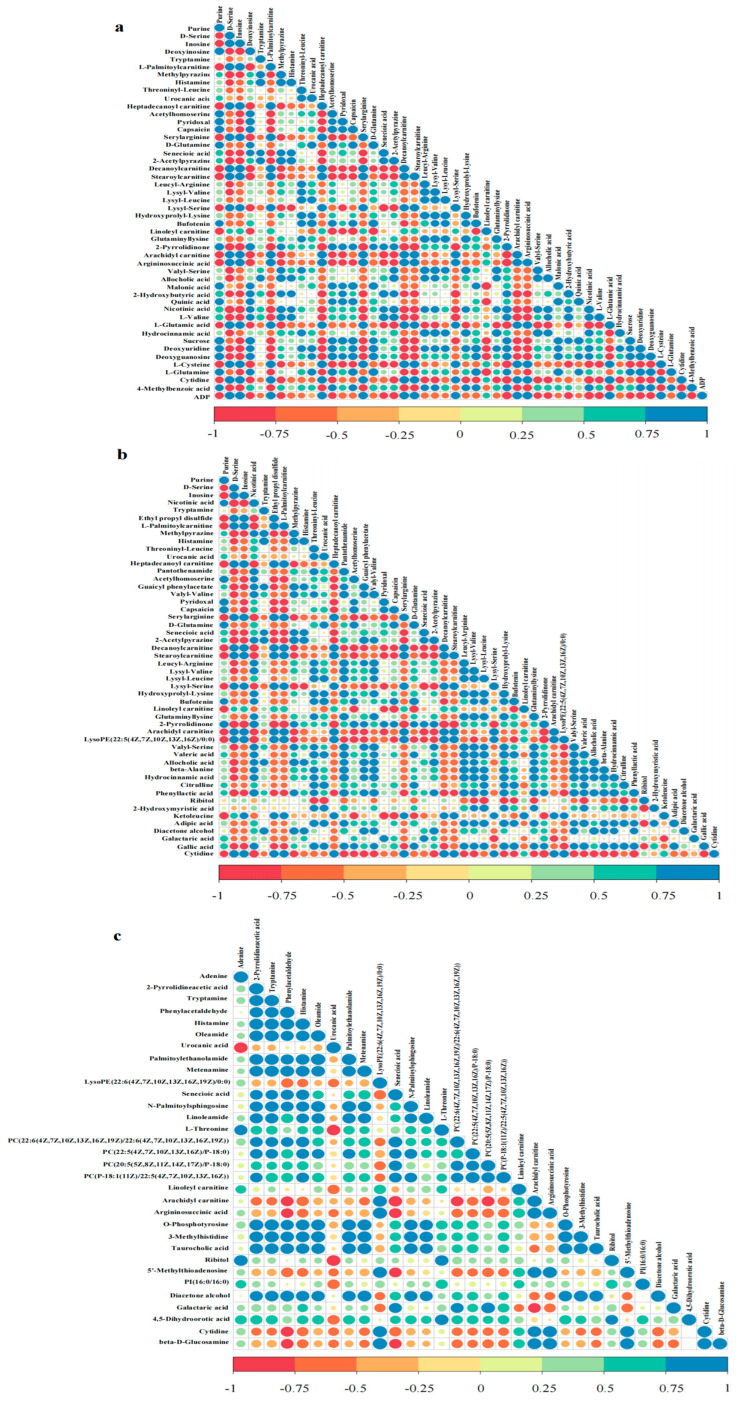
Correlation maps of metabolites produced during different fermentation periods of mandarin fish: (**a**) 0D versus 4D; (**b**) 4D versus 8D; and (**c**) 8D versus 12D.

As shown in Figure 3, in the 0D versus 4D group, inosine and lysyl-serine were negatively correlated with L-valine, senecioic acid, and 2-hydroxybutyric acid and positively correlated with L-palmitoylcarnitine and heptadecanoyl carnitine. In the 4D versus 8D group, lysyl-serine and arachidyl carnitine were positively correlated with L-palmitoylcarnitine and cytidine and negatively correlated with senecioic acid and nicotinic acid. In the 8D versus 12D group, argininosuccinic acid and cytidine were negatively correlated with phenylacetaldehyde and senecioic acid and positively correlated with linoleyl carnitine and arachidyl carnitine, whereas L-threonine was positively correlated with histamine and 3-methylhistidine. Histamine was consistently positively correlated with tryptamine and negatively correlated with cytidine and arachidyl carnitine throughout the fermentation process, whereas arachidyl carnitine was positively correlated with linoleyl carnitine.

### 3.4. Cluster Analysis of Differential Metabolites

**Figure 4:**
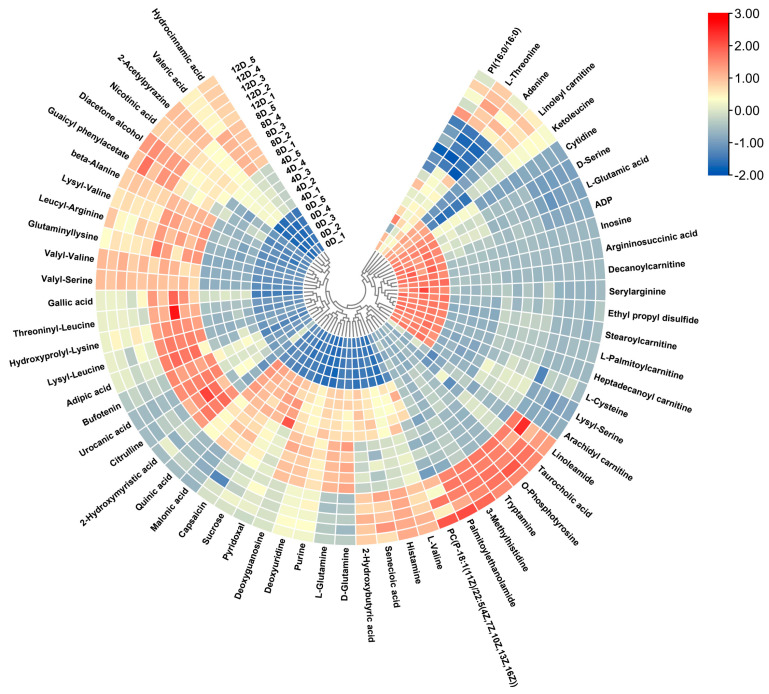
Heatmap of hierarchical cluster analysis of mandarin fish samples at different fermentation stages.

The above three sets of data were collated, and 62 differential metabolites were identified, including 11 amino acids, 11 short peptides, 11 fatty acids, 3 alkaloids, and 4 organic acids. The results of the hierarchical clustering analysis of the differential metabolites are shown in Figure 4. The color blocks in different positions represent the magnitude of the correlation coefficient between the corresponding metabolites, with red indicating a positive correlation and blue a negative correlation—the darker the color, the stronger the correlation. The analysis revealed that the metabolite abundances varied considerably between different fermentation periods, and the metabolites that abundantly increased in different fermentation periods were clustered into one cluster. This distinguished the samples from different fermentation periods.

#### 3.4.1. Amino Acids

The concentration of amino acids considerably changed as the fermentation of mandarin fish proceeded, and most amino acids accumulated with an increase in fermentation time. The concentration of L-glutamic acid decreased at the beginning of fermentation, whereas those of L-glutamine and D-glutamine gradually increased, reaching maximum values on day 8 of fermentation. This could be because some microorganisms metabolize nutrients during the fermentation of mandarin fish to produce acetic acid and butyric acid, which makes fermented samples acidic [24]. Additionally, under the action of glutamine synthetase, NH_4_^+^ and glutamic acid are converted to glutamine [25]. Glutamic acid is a fresh-tasting amino acid and the main substance responsible for the fresh taste of fermented fish. The concentrations of sweet amino acids, L-threonine and beta-alanine, gradually increased in the later stages of fermentation, whereas those of D-serine gradually decreased as the fermentation progressed. The concentrations of L-threonine and beta-alanine increase significantly in the later stages of fermentation in carp, and these amino acids are the main contributors to the flavor [26]. This was consistent with the changes observed during the fermentation of mandarin fish. L-Valine, a bitter amino acid, reached its maximum abundance on day 12 of fermentation. The higher concentration of bitter amino acids did not affect the overall taste of mandarin fish, as the taste had an ablative effect. The elimination of bitter and sweet amino acids reduces the bitterness and sweetness. Furthermore, the addition of NaCl to a bitter-sweet mixture inhibits the bitterness and enhances the sweetness by releasing a bitter taste-inhibiting component of the mixture [27]. Therefore, the sweet amino acid concentration increased considerably during the later stages of fermentation.

#### 3.4.2. Small Peptides

In addition to amino acids, various small peptides were produced during the fermentation of mandarin fish. Short peptides are superior to amino acids in both absorption rate and biological function [28]. Peptides with relatively small molecular weights impart sweet, sour, salty, fresh, and bitter flavors, and enrich and improve the sensory characteristics of fermented products [29]. The concentrations of lysyl-serine, seryl-arginine, and argininosuccinic acid decrease in the early stages of fermentation as reported by a study on *Aspergillus*, probably because of the dominance of *Aspergillus* early in fermentation, which degrades small peptides [4]. The concentrations of six bitter peptides (valyl-valine, leucyl-arginine, lysyl-leucine, threoninyl-leucine, lysyl-valine, and valyl-serine) significantly increased on day 8 of fermentation, but the concentrations of lysyl-leucine and threoninyl-leucine gradually decreased after this point. Bitter peptides are the main cause of bitterness; however, the increase in the bitter peptide concentration does not affect the overall taste of fermented mandarin fish, as the freshness amino acid concentration reaches its maximum on day 8 of fermentation and inhibits the bitterness in fermented fish [30]. Zhao et al. [31] found that Glu-Leu and Glu-Thr contribute to the freshness flavor of dry-cured fish, and γ-glutamyl-valyl-glycine, a peptide found in fish sauce, produces a strong taste and can improve the flavor of food [32]. Therefore, the accumulation of small peptides can improve the nutritional quality and flavor of fermented mandarin fish.

#### 3.4.3. Fatty Acid Class

The formation of most flavor compounds is generally associated with the degradation of fatty acids [33]. The concentrations of linoleyl carnitine, arachidyl carnitine, decanoylcarnitine, L-palmitoylcarnitine, stearoylcarnitine, and heptadecanoyl carnitine were the maximum on day 0 of fermentation and decreased sharply as fermentation proceeded, probably due to abundant growth of microorganisms at the beginning of fermentation, which led to the degradation of these fatty acids into energy and carbon for microbial growth. A similar decrease in the fatty acid concentration was observed in the early stages of fermentation of shrimp paste [34]. On day 8 of fermentation, 2-hydroxymyristic acid, valeric acid, and adipic acid were produced again, and their concentrations gradually decreased thereafter. Adipic acid is a dicarboxylic acid with a soft and persistent acidity that acts as a good acidity regulator [35]. The adipic acid concentration reached its maximum on day 8 of fermentation, which could suppress bitterness during fermentation and regulate the taste of mandarin fish to some extent.

#### 3.4.4. Other Compounds

Organic acids are among the most important flavor compounds in aquatic food-products, which mainly confer an acidic flavor to foods. Organic acids in meat products help stabilize and enhance the quality and improve the flavor. Ketoleucine levels continuously decreased at the beginning of fermentation, whereas the malonic acid, 2-hydroxybutyric acid, and palmitoylethanolamide levels increased substantially during fermentation, increasing the flavor of fermented mandarin fish. Malonic acid is a part of the biosynthetic route of phenolic compounds and is involved in the biosynthesis of fatty acids [36]. Gallic acid is an aromatic compound that exerts significant antioxidant effects by preventing the primary and secondary oxidation of lipids and scavenging free radicals [37]. As shown in Figure 4, the gallic acid concentration increased pre-fermentation, which was negatively correlated with the overall trend of changes in the fatty acid concentration. The nicotinic acid concentration increased throughout fermentation. Nicotinic acid maintains normal metabolism in the body, thereby preventing skin inflammation and pigmentation development, and is closely related to protein, fat, and carbohydrate metabolism [38].

### 3.5. Quantitative Analysis of Free Amino Acids during Different Stages in the Fermentation of Mandarin Fish

**Figure 5:**
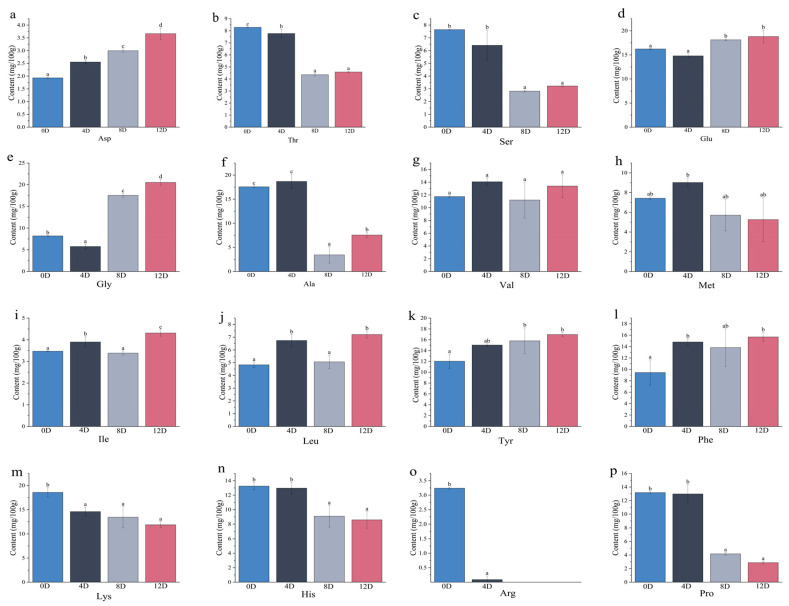
Concentrations of different free amino acids of mandarin fish at different stages of fermentation. Labels on the graph represent significant differences (*p* < 0.05). (**a**): Arginine, (**b**): Threonine, (**c**): Serine, (**d**): Glutamic acid, (**e**): Glycine, (**f**): Alanine, (**g**): Valine, (**h**): Methionine, (**i**): Isoleucine, (**j**): Leucine, (**k**): Tyrosine, (**l**): Phenylalanine, (**m**): Lysine, (**n**): Histidine, (**o**): Arginine, (**p**): Proline.

The free amino acids were quantified during different stages of fermentation to further investigate the key taste compounds that are responsible for the flavor of fermented mandarin fish. Sixteen free amino acids were found in all samples. The concentration of the total free amino acids reached a maximum of 160.17 mg/100 g on day 4 of fermentation; glutamic acid, glycine, isoleucine, leucine, tyrosine, and phenylalanine reached maximum levels on day 12 of fermentation; and the concentrations of lysine, histidine, proline, and arginine continuously decreased as the fermentation progressed, with arginine having been degraded on day 8 of fermentation (Figure 5).

Amino acids are vital taste-facilitating substances that confer sweetness, freshness, and bitterness to foods [39]. Sweet, umami, and bitter amino acids accounted for 46.80%, 11.59%, and 41.64% of the total amino acid concentration, respectively, in the initial fermentation stage. The concentration of sweet amino acids decreased by 31.07% on day 12 of fermentation compared with that at the beginning of fermentation, and the concentrations of umami and bitter amino acids increased by 23.73% and 9.13%, respectively. The increase in the concentrations of bitter and umami amino acids in the late stage of fermentation of mandarin fish are consistent with the results of Yang et al. [40].

Alanine, lysine, and glycine contributed to the sweetness of mandarin fish during fermentation, and the sweetness exhibited by alanine and glycine had a synergistic effect on the umami taste [41]. Glutamic acid and aspartic acid are both fresh-tasting amino acids, among which aspartic acid can enhance the salty taste and mask the bitter taste in fermented products [42]. Glutamic acid, a component of monosodium glutamate, plays an important role in the freshness flavor of food. The concentration of glutamic acid was significantly higher than that of aspartic acid on day 12 of fermentation, indicating a greater contribution of glutamic acid to the fresh flavor of mandarin fish. Val and His are relatively bitter amino acids. The freshness flavor combines sweet, salty, and bitter flavors that play an important role in consumer satisfaction [43]. Therefore, the high concentrations of bitter and sweet amino acids in mandarin fish had a positive effect on the fermentation and production of the umami flavor.

### 3.6. Pathway Analysis of Differential Metabolites

**Figure 6:**
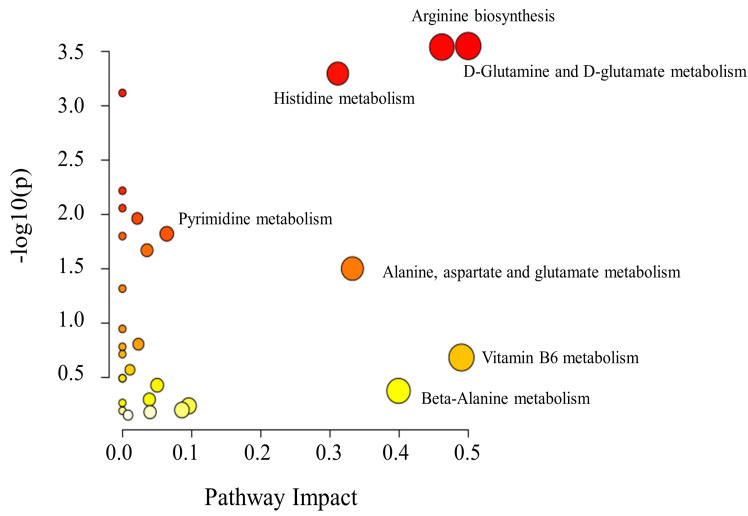
Pathway analysis diagram. Each bubble represents a metabolic pathway. The horizontal coordinates and the size of the bubble correspond to the influence factor of the pathway in the topological analysis.

To identify the metabolic pathways with the highest relevance to metabolite differences during the fermentation of mandarin fish, enrichment analysis and topological structure analysis of differential metabolites were performed using the KEGG database. Twenty-eight metabolic pathways were identified (Figure 6), which mainly involved amino acid metabolism, fatty acid metabolism, and vitamin metabolism pathways. Some pathways were significant—D-glutamine, D-glutamate, beta-alanine, histidine, alanine, aspartate, glutamate, and vitamin B6 metabolism pathways, as well as the arginine biosynthesis pathway.

Pathways associated with D-glutamine, D-glutamate, and vitamin B6 metabolism and arginine biosynthesis were found to have greater effects on the production of metabolites during fermentation than metabolic pathways such as histidine, alanine, and aspartate (Figure 6). Metabolites produced through the D-glutamine and D-glutamate metabolic pathways were significantly enriched.

Most of the 28 metabolic pathways identified in this study were amino acid metabolic pathways, with the most important changes observed in the arginine biosynthesis and beta-alanine metabolism pathways. These pathways involve many derivatives, such as glutamine, citrulline, aspartic acid, arginine, ornithine, histidine, and lysine, and the metabolism of histidine leads to the production of glutamic acid. Thus, these amino acid metabolic pathways play important roles in the flavor formation of mandarin fish, especially in the production of glutamic acid, which plays an important role in the freshness flavor of mandarin fish.

## 4. Conclusions

The current study sheds light on the taste-related metabolite profiles during the fermentation of mandarin fish based on UHPLC-QE-MS/MS untargeted metabolomics. Sixty-two metabolites were identified, including amino acids, small peptides, fatty acids, alkaloids, and organic acids. Furthermore, the quantification of free amino acids indicated that these metabolites, especially glutamic acid and aspartic acid, had a considerable influence on the taste of fermented mandarin fish by altering the fresh, bitter, and sweet flavors. The metabolic pathways with significant effects on metabolites included arginine biosynthesis and the D-glutamine, D-glutamate, beta-alanine, histidine, alanine, aspartate, glutamate, and vitamin B6 metabolism pathways. These findings provide a better understanding of the metabolite composition and overall taste profile of mandarin fish during fermentation, as well as important information that may help improve the taste of fermented mandarin fish.

## Data Availability

Not applicable.
